# An algorithm for computing Schubert varieties of best fit with applications

**DOI:** 10.3389/frai.2023.1274830

**Published:** 2023-11-24

**Authors:** Karim Karimov, Michael Kirby, Chris Peterson

**Affiliations:** Department of Mathematics, College of Natural Sciences, Colorado State University, Fort Collins, CO, United States

**Keywords:** Schubert variety of best fit, manifold approximation, subspace classification, geometry of learning, neural network, abstract node, GPU parallel computing

## Abstract

We propose the geometric framework of the Schubert variety as a tool for representing a collection of subspaces of a fixed vector space. Specifically, given a collection of *l*-dimensional subspaces *V*_1_, …, *V*_*r*_ of ℝ^*n*^, represented as the column spaces of matrices *X*_1_, …, *X*_*r*_, we seek to determine a *representative* matrix *K*∈ℝ^*n*×*k*^ such that each subspace *V*_*i*_ intersects (or comes close to intersecting) the span of the columns of *K* in at least *c* dimensions. We formulate a non-convex optimization problem to determine such a *K* along with associated sets of vectors {*a*_*i*_} and {*b*_*i*_} used to express linear combinations of the columns of the *X*_*i*_ that are close to linear combinations of the columns of *K*. Further, we present a mechanism for integrating this representation into an artificial neural network architecture as a computational unit (which we refer to as an abstract node). The representative matrix *K* can be learned *in situ*, or sequentially, as part of a learning problem. Additionally, the matrix *K* can be employed as a change of coordinates in the learning problem. The set of all *l*-dimensional subspaces of ℝ^*n*^ that intersects the span of the columns of *K* in at least *c* dimensions is an example of a Schubert subvariety of the Grassmannian *GR*(*l, n*). When it is not possible to find a Schubert variety passing through a collection of points on *GR*(*l, n*), the goal of the non-convex optimization problem is to find the Schubert variety of best fit, i.e., the Schubert variety that comes as close as possible to the points. This may be viewed as an analog of finding a subspace of best fit to data in a vector space. The approach we take is well-suited to the modeling of collections of sets of data either as a stand-alone Schubert variety of best fit (SVBF), or in the processing workflow of a deep neural network. We present applications to some classification problems on sets of data to illustrate the behavior of the method.

## 1 Introduction

A variety of powerful tools have been developed in Machine Learning and Artificial Intelligence and these have led to remarkable applications. A damper on the success of these tools is the fact that the resulting models are frequently difficult to explain and the predictions may not be trustworthy in high stakes scenarios, e.g., those related to medical diagnoses, battlefield scenarios, or intelligence gathering. One reason the success of ML/AI tools is challenging to explain, or trust, is that these models were designed, first and foremost, to make accurate predictions; attempts to interpret or explain the effectiveness of the models being only an afterthought.

In this paper, we propose a methodology based on an interpretable mathematical framework, i.e., the geometric setting of the Schubert Variety, as the starting point, and explore variations on the theme to determine optimal architectures for predictive modeling. The goal of this research is to begin with a mathematically motivated explainable approach, and then optimize its performance through numerical algorithms. Optimistically, this approach will lead to results of comparable accuracy of the traditional ML/AI toolkit, however, with the advantage of explainability and trustworthiness. To this end, we propose an approach for characterizing sets of linear spaces, i.e., fitting/approximating subspaces with one, or more representative spaces. Additionally, we demonstrate how the mathematical framework and resulting algorithms can be integrated into current tools including deep feed forward neural networks.

The initial development of ML/AI, focused more on thinking machines than interpretability. Human intelligence, and the associated architecture of the human brain, have been a driving force in biomimetic approaches. For example, the McCullough-Pitts node (McCulloch and Pitts, 1943) and its associated weights were proposed as a mathematical model of the cell and its associated neurons, respectively. The first transfer function at a computational node was a simple step function replicating the firing or quiescence of a neuron. Impressively, arrays of such networks were shown to be able to serve as models of associative memory, and to even recall patterns which were partially occluded (Hertz et al., 1991). Hebbian learning (Hebb, 1949) was proposed as a model for memory and convincingly analyzed as a dynamical system where the patterns were stored as fixed points (Hertz et al., 1991), an early appearance of the application of mathematical analysis for explainability of artificial neural networks.

Geometric ideas emerged with Rosenblatt's simple perceptron (Rosenblatt et al., 2002), still loosely based on neurons firing, where the dot product operation between a pattern and a weight vector gave rise to classification via the interpretation of the models as splitting a space into two half-spaces. The multilayer perceptron extended these ideas in natural ways, however, at the expense of geometric interpretability. Nonlinear data reduction was made possible by autoencoder neural networks (Kramer, 1991; Oja, 1992). The encoder-decoder architecture has been widely exploited by Variational Autoencoders (Kingma and Welling, 2019), Centroid-Encoders (Ghosh and Kirby, 2022), and Transformers (Vaswani et al., 2017). These developments, while providing powerful tools, widely lack mathematical underpinnings that provide insight into their utility.

**Figure 1:**
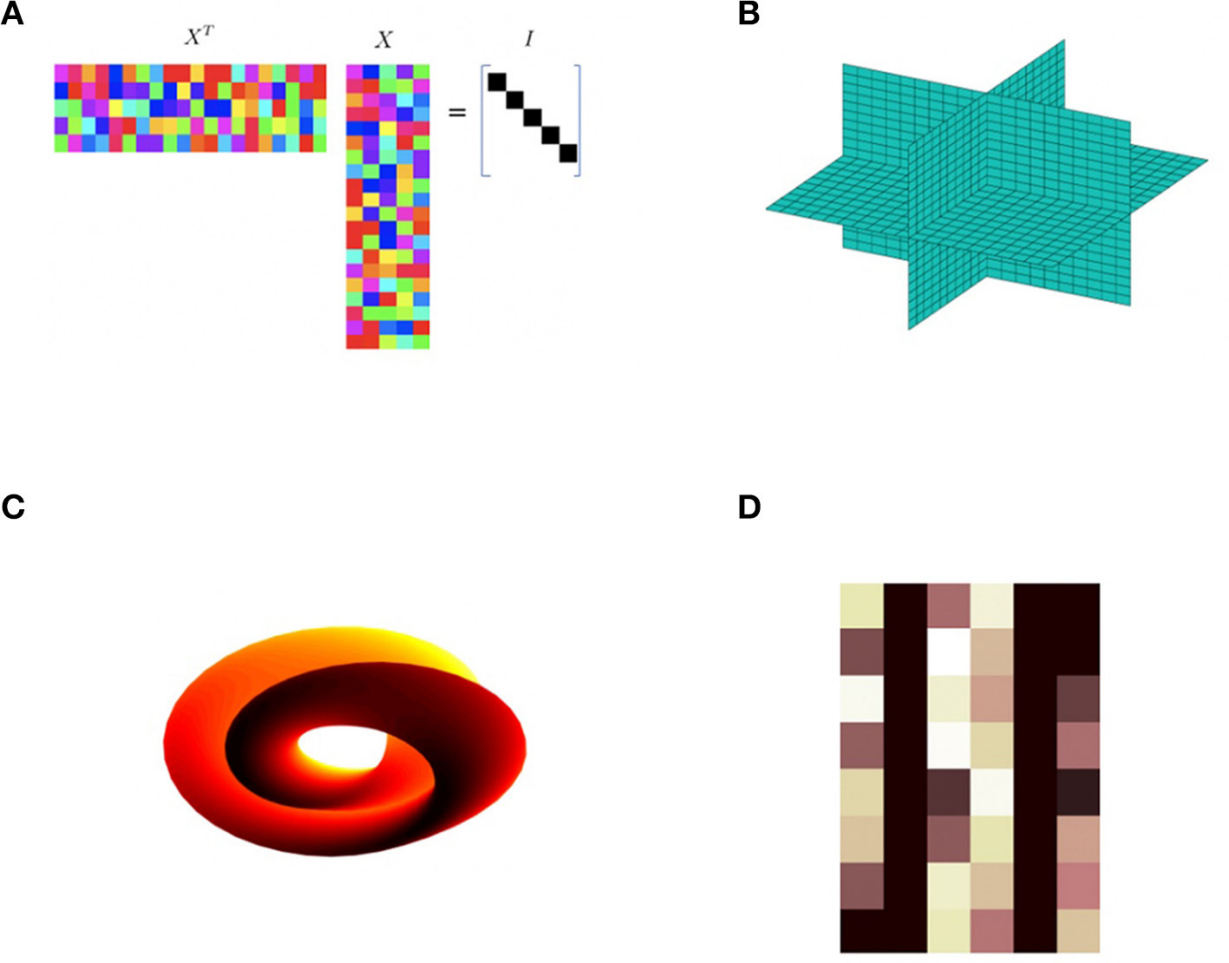
Various examples of abstract nodes. These *computational units* can be integrated into data fitting problems, e.g., multilayer neural networks, to extract topological or geometric structure. This paper focuses on the particular case of the Schubert variety constraint **(D)**. **(A)** This computational unit with matrix values *X* is required to satisfy *X*^*T*^*X* = 1. **(B)** This unit requires the data to reside in a union of sub-spaces such as the *xy*, *xz*, and *yz* planes shown. **(C)** The Klein bottle is an example of a unit manifold constraint. **(D)** A Schubert variety constraint.

In this paper, we illustrate how one can use mathematical theory and geometric frameworks as a design philosophy in the construction of novel neural network architectures. This general idea can be found in previous work, e.g., geometric, or topological nodes such as circular (Kirby and Miranda, 1996), or spherical computational units (Hundley et al., 1995). Whitney's theorem has also been invoked to provide a basis to understand the power of autoencoders from a geometric perspective (Broomhead and Kirby, 2000) and to provide insights into novel architectures (Broomhead and Kirby, 2001) and dimension estimation (Anderle et al., 2002; Kvinge et al., 2018a,b). These ideas are central to the computation of homeomorphisms between *data sets* residing in spaces of differing dimensions. Using these ideas as motivation we can envision extending the concept of an abstract node more generally to algebraic varieties related to Generalized Principal Component Analysis (Vidal et al., 2005), Klein bottles, Grassmannians, and Schubert Varieties (see Figure 1).

In this paper, we focus our attention on the mathematical framework of the Schubert Variety described in what follows. We are motivated by the idea that a Schubert variety is to a Flag or Grassmann manifold what a subspace is to a vector space. Flag manifolds, and their special case the Grassmann manifolds, are examples of homogeneous manifolds particularly relevant to and amenable to subspace methods in Data Science. They have been observed to be particularly robust to data collected under variations in pattern state (and indeed exploit structure in such data sets). For example, digital images of an object, collected under variations in illumination, are known to sweep out a convex cone. If the object is Lambertian then this cone has been shown to lie close to a low dimensional linear space which can in turn be represented by a point on a Grassmannian (Beveridge et al., 2008). The flag manifold comes equipped with geometric features capable of representing sets of data where the number of points is larger than the number of dimensions in the ambient space (Ma et al., 2021). We note that the flag mean proposed in Marrinan et al. (2014, 2015) and Mankovich et al. (2022), and its various extensions, are a special case of the work proposed here.

Briefly, in this paper we propose several optimization problems which are used to produce a geometric object, e.g., a Schubert variety of best fit (SVBF), that optimally represents a set of linear subspaces of a fixed vector space ℝ^*n*^. In several applications, the set of linear spaces are obtained from sets of sets of data. The optimization problem is determined by a real valued objective function on a manifold (typically a Grassmann or Flag manifold) whose points parameterize a family of potential Schubert varieties of best fit. Further, we show how this framework can be viewed as a component of a machine learning architecture integrated, e.g., into the broader framework of feed forward neural networks.

This paper is organized as follows: Section 2 presents a brief overview of a class of manifolds built from matrix group actions. In Section 3, we describe Schubert varieties and how to define a Schubert variety of best fit to a collection of subspaces. Section 4 describes a specific implemented optimization problem for finding a Schubert variety of best fit and applies it to an illustrative example. Section 5 provides three algorithms and shows how to implement the ideas as an abstract node. Lastly, in Section 6, we provide concluding remarks summarizing the overall findings and contributions of our research.

## 2 Background

Consider the set, *S*, of *n*×*n* invertible matrices whose inverse is equal to its transpose, i.e., $S=\left\{A\in {\mathbb{R}}^{n\times n}\;|\;A^{T}A=I_{n}\right\}$. *S* contains the identity matrix, *I*_*n*_, and is closed under the operations of matrix inversion and matrix multiplication. In other words, if *A* and *B* are in *S* then both *A*^−1^ and *AB* are in *S*. The set *S* together with the binary operation of matrix multiplication is known as the *orthogonal group* *O*(*n*). Alternatively, *O*(*n*) is the group of distance-preserving transformations of an *n*-dimensional Euclidean space that preserve a fixed point. A distinguished subgroup of *O*(*n*) is the special orthogonal group *SO*(*n*) consisting of elements of *S* with determinant equal to one (orientation preserving transformations). From a geometric perspective, the orthogonal group *O*(*n*) can be considered as a manifold whose points parameterize ordered orthonormal bases of ℝ^*n*^. As a manifold, it consists of two connected components corresponding to the square orthogonal matrices with determinant +1 and those with determinant −1. Its dimension as a real manifold is (n2). Several interesting manifolds can be built, through a “quotient” operation, by considering the action of subgroups of *O*(*n*) [resp. *SO*(*n*)] on *O*(*n*) [resp. *SO*(*n*)] through multiplication. Some particularly relevant examples are the following:

- Grassmann manifolds *GR*(*l, n*)
- Oriented Grassmann manifolds $\tilde{GR}\left(l,n\right)$
- Flag manifolds *FL*(*l*_1_, *l*_2_, ..., *l*_*m*_; *n*)
- (Partially) oriented flag manifolds
- Steifel manifolds *ST*(*l, n*)

**Grassmann manifold**—The Grassmannian of *l*-dimensional subspaces of *R*^*n*^ is denoted by *GR*(*l, n*). Points on *GR*(*l, n*) correspond to *l*-dimensional subspaces of ℝ^*n*^. It can be built as a coset space *O*(*n*)/*O*(*l*) × *O*(*n*−*l*) where *O*(*l*) × *O*(*n*−*l*) denotes *n*×*n* matrices consist of an *l*×*l* orthogonal block and an *n*−*l*×*n*−*l* orthogonal block. Through this identification, points on *GR*(*l, n*) correspond to equivalences classes of *n*×*n* orthogonal matrices where two such matrices are identified if the span of their first *l* columns agree. We can also think of points on *GR*(*l, n*) as corresponding to equivalences classes of *n*×*l* orthogonal matrices where two such matrices are identified if they have the same column space. *GR*(*l, n*) can be considered as a homogeneous space and as a differentiable manifold. As a real manifold, the dimension of *GR*(*l, n*) is *l*(*n*−*l*) = *dim*(*O*(*n*))−*dim*(*O*(*l*))−*dim*(*O*(*n*−*l*)).

**Oriented Grassmann manifold**—The oriented Grassmannian of all *oriented* *l*-dimensional subspaces of ℝ^*n*^ is denoted $\tilde{GR}\left(l,n\right)$. It can be built as a coset space *SO*(*n*)/*SO*(*l*) × *SO*(*n*−*l*). There is a natural 2:1 covering map from $\tilde{GR}\left(l,n\right)$ to *GR*(*l, n*). A special case is $\tilde{GR}\left(1,n\right)$ whose cosets correspond to points on the *n*−1 dimensional sphere *S*^*n*−1^ in *R*^*n*^. *GR*(1, *n*) corresponds to the real projective space *RP*^*n*−1^. *RP*^*n*−1^ can also be built from *S*^*n*−1^ by identifying antipodal points on the sphere. In the map from $\tilde{GR}\left(1,n\right)$ to *GR*(1, *n*), a pair of antipodal points on the sphere get mapped to a single point in projective space. As a real manifold, the dimension of $\tilde{GR}\left(l,n\right)$ is the same as the dimension of *GR*(*l, n*).

**Flag manifold**—*FL*(*l*_1_, *l*_2_, ..., *l*_*m*_; *n*) = collection of all flags of the form $V_{1}\subset V_{2}\subset \cdots \subset V_{m}\subset {\mathbb{R}}^{n}$ such that dim*V*_*i*_ = *l*_*i*_. The flag manifolds can be built by considering quotients of *O*(*n*) by a direct product of smaller orthogonal groups. More precisely by the quotient of *O*(*n*) by *O*(*l*_1_) × *O*(*l*_2_−*l*_1_) × *O*(*l*_*m*_−*l*_*m*−1_) × *O*(*n*−*l*_*m*_). As a real manifold, the dimension of *FL*(*l*_1_, *l*_2_, ..., *l*_*m*_; *n*) is *dim*(*O*(*n*))−*dim*(*O*(*l*_1_))−*dim*(*O*(*l*_2_−*l*_1_))−*dim*(*O*(*l*_3_−*l*_2_))−⋯−*dim*(*O*(*l*_*m*_−*l*_*m*−1_))−*dim*(*O*(*n*−*l*_*m*_)).

**Oriented flag manifold**—$FL^{•}\left(l_{1},l_{2},...,l_{m};n\right)$ = collection of all oriented flags of the form $V_{1}\subset V_{2}\subset \cdots \subset V_{m}\subset {\mathbb{R}}^{n}$ such that dim*V*_*i*_ = *l*_*i*_. The oriented flag manifolds can be built by considering quotients of *SO*(*n*) by a direct product of smaller special orthogonal groups. There is a natural 2^*m*^:1 covering map from $FL^{•}\left(l_{1},l_{2},...,l_{m};n\right)$ to *FL*(*l*_1_, *l*_2_, ..., *l*_*m*_; *n*). As a real manifold, the dimension of $FL^{•}\left(l_{1},l_{2},...,l_{m};n\right)$ is the same as the dimension of *FL*(*l*_1_, *l*_2_, ..., *l*_*m*_; *n*).

**Partially oriented flag manifold**—The partially oriented flag manifolds can be built by considering quotients of *SO*(*n*) by a direct product of a mixture of smaller special orthogonal groups and smaller orthogonal groups [with the additional constraint that the direct product is a subgroup of *SO*(*n*)]. There are many different types of partially oriented flag manifolds.

**Steifel manifold**—Points on the Steifel manifold *ST*(*l, n*) correspond to ordered orthonormal sets of *l* vectors in ℝ^*n*^. Alternatively, points on *ST*(*l, n*) correspond to elements in the set $\left\{A\in R^{n\times l}|A^{T}A=I_{l}\right\}$. The Steifel manifold *ST*(*l, n*) can also be considered as the oriented flag manifold *FL*°(1, 2, 3, …, *l*; *n*). The dimension of *ST*(*l, n*) is (*n*−1) + (*n*−2)+⋯+(*n*−*l*). There is a natural 2^*l*^:1 covering map from *ST*(*l, n*) to *FL*(1, 2, …, *l*; *n*).

The homogeneous spaces listed above are all compact differentiable manifolds whose points parameterize flags of (oriented) subspaces of *R*^*n*^ with a common signature *l*_1_, …, *l*_*m*_. Many problems of interest can be formulated in terms of optimizing some function on one or several of these or related parameter spaces. The formulation and solution is often driven by geometric considerations. Problems which have an efficient numerical solution are particularly appealing. Some additional parameter spaces of interest include Affine space, Euclidean space, Hyperbolic space, Anti-di Sitter space, and product spaces built out of any combination of these spaces, their oriented versions, or any the previously described homogeneous spaces from above.

## 3 Schubert varieties

A Schubert variety in a Grassmann or flag manifold is a certain kind of subvariety (typically with singularities) that can be defined by a collection of linear algebraic incidence constraints with respect to a fixed flag, *F*, drawn from some flag variety *FL*(*k*_1_, *k*_2_, …, *k*_*m*_; *n*). If you vary the flag then you vary the Schubert variety. The Schubert variety can be viewed as a kind of moduli space while points on the flag variety can be seen as parameterizing a family of Schubert varieties of a particular type.

### 3.1 Definition of Schubert variety

We first consider an example of a kind of Schubert variety that will be referred to several times in this paper. If *W*∈*GR*(*k, n*) then *W* is a rank *k* subspace of ℝ^*n*^. Given a pair of non-negative integers (*c, l*), we can define a collection of points in *GR*(*l, n*) by


$$\Omega_{c,k,l}\left(W\right)=\left\{V\in GR\left(l,n\right)\;|\;\dim\left(V\cap W\right)\geq c\right\}$$

In order for this set of points to be nonempty we will need that *c* ≤ *l* and that *c* ≤ *k*. For each *W*∈*GR*(*k, n*), Ω_*c, k, l*_(*W*) is a subvariety of *GR*(*l, n*). As a consequence, *GR*(*k, n*) can be seen as parameterizing a family of such subvarieties of *GR*(*l, n*). The subvariety Ω_*c, k, l*_(*W*) is an example of a particular kind of Schubert variety on *GR*(*l, n*). As mentioned in the previous paragraph, Schubert varieties are typically singular.

The example in the previous paragraph can be extended in several directions. The following is an example where *W* is drawn from a more general Flag manifold. Recall that *FL*(*k*_1_, *k*_2_, ..., *k*_*m*_; *n*) is the collection of all flags of the form $W_{1}\subset W_{2}\subset \cdots \subset W_{m}\subset {\mathbb{R}}^{n}$ such that dim*W*_*i*_ = *k*_*i*_. To emphasize that *W* is a flag of vector spaces, we will write *W* as **W**. We call *FL*(*k*_1_, *k*_2_, ..., *k*_*m*_; *n*) an *m* step flag manifold. Points on this manifold are *m* step flags of signature (*k*_1_, *k*_2_, …, *k*_*m*_). A Grassmann manifold is a one step flag manifold. For instance, *GR*(*k, n*) = *FL*(*k*; *n*). A large collection of Schubert subvarieties of *GR*(*l, n*) can be built as follows: Pick a point **W** on a flag manifold *FL*(*k*_1_, *k*_2_, ..., *k*_*m*_; *n*) and an *m*-tuple $\vec{c}=\left(c_{1},\ldots ,c_{m}\right)$ (thus **W** corresponds to a specific flag *W*_1_⊂*W*_2_⊂⋯⊂*W*_*m*_ where dim*W*_*i*_ = *k*_*i*_). Let $\vec{k}=\left(k_{1},k_{2},...,k_{m}\right)$. An associated subvariety of *GR*(*l, n*) is given by


$$\Omega_{\vec{c},\vec{k},l}\left(W\right)=\left\{V\in GR\left(l,n\right)\;|\;\dim\left(V\cap W_{i}\right)\geq c_{i}\text{ for }1\leq i\leq m\right\}$$

Points on *FL*(*k*_1_, *k*_2_, ..., *k*_*m*_; *n*) parameterize a family of such subvarieties.

In a similar manner, one can build subvarieties of a more general flag manifold *FL*(*l*_1_, …, *l*_*s*_; *n*). The subvarieties will be written $\Omega_{C,\vec{k},\vec{l}}\;\left(W\right)$. The data that is determining the subvariety, $\Omega_{C,\vec{k},\vec{l}}\;\left(W\right)$, is the space from which we draw our fixed flags [e.g., **W**∈*FL*(*k*_1_, …, *k*_*m*_; *n*)], a space on which the subvariety lives [e.g., *FL*(*l*_1_, …, *l*_*s*_; *n*)], and incidence constraints, *c*_*i, j*_, stored in an *m*×*s* array *C*. We have


$$\begin{array}{l} \;\Omega_{C,\vec{k},\vec{l}}\;\left(W\right)=\\ \{V\in FL(\vec{l};n)\;|\;\dim(V_{j}\cap W_{i})\geq C_{i,j}\text{ for }1\leq i\leq m,\;1\leq j\leq s\}. \end{array}$$

### 3.2 Schubert varieties of best fit

Suppose we are given a collection of *l*-dimensional subspaces *D* = {*V*_1_, …, *V*_*r*_} of ℝ^*n*^. Each element in *D* can be thought of as a point in the Grassmannian *GR*(*l, n*) thus we have *r* points on *GR*(*l, n*). We seek to determine a Schubert variety of best fit to the *r* points. In order for this problem to make sense, we need to answer two questions: the first is “What class of Schubert varieties are you going to use to best fit the data?” and the second question is “What is the objective function you are trying to optimize when searching for a Schubert variety of best fit?.” Intuitively, we are searching for a Schubert variety that comes as “close as possible” to the set of points determined by *D*. This should remind you of finding a “linear space of best fit” to a set of points in ℝ^*n*^. For our purposes, given a point or collection of points on *Gr*(*l, n*) and a Schubert variety *S*, we further seek to have a measurement of closeness that is orthogonally invariant, i.e., is invariant to the action of the orthogonal group *O*(*n*). Points on a Grassmannian correspond to subspaces and Schubert varieties are defined in terms of incidence conditions with respect to a fixed flag of subspaces. With this in mind, in order to achieve measurements that are orthogonally invariant, it is natural to write the measurement of closeness in terms of principle angles between the subspaces involved. There are many different ways in which this can be carried out and this leads to many different answers to the problem.

In what follows, let *D* = {*V*_1_, *V*_2_, …, *V*_*r*_} be a collection of *l* dimensional subspaces considered as points on *GR*(*l, n*). Given positive integers *k* and *c*, our goal is to find a point *W*∈*GR*(*k, n*) such that the Schubert variety


$$\Omega_{c,k,l}\left(W\right)=\left\{V\in GR\left(l,n\right)\;|\;\dim\left(V\cap W\right)\geq c\right\}$$

comes as close as possible to the points in *D*. We will break this up into three parts. The first part will be to define a measurement of closeness, *d*[*V*_*i*_, Ω_*c, k, l*_(*W*)], between a single point *V*_*i*_∈*GR*(*l, n*) and the Schubert variety Ω_*c, k, l*_(*W*) [with *W*∈*GR*(*k, n*)]. The second part will be to combine these single point measurements into a measurement of closeness between a set of points *D* = {*V*_1_, *V*_2_, …, *V*_*r*_}⊂*GR*(*l, n*) and the Schubert variety Ω_*c, k, l*_(*W*). The third part will be to find a W^*^∈*GR*(*k, n*) that optimizes this measure of closeness.

With respect to the third part, suppose we have fixed a real valued measurement of closeness between a set of points, *D*⊂*GR*(*l, n*) and a Schubert variety Ω_*c, k, l*_(*W*). For each point *W*∈*GR*(*k, n*) we have effectively assigned a real number (the closeness measure) thus we have a function *F*:*GR*(*k, n*) → ℝ. Since *GR*(*k, n*) is a compact manifold, this real valued function will attain both its maximum and minimum values. Our goal is to describe an algorithm, implemented in a neural network, to find a point in *GR*(*k, n*) that achieves either a maximum or a minimum of this function.

### 3.3 Examples of distance/closeness measures

Let *V*_1_, *V*_2_ be subspaces of ℝ^*n*^ of dimension *l*. Recall that the principal angles between *V*_1_ and *V*_2_ satisfy 0 ≤ θ_1_ ≤ θ_2_ ≤ ⋯ ≤ θ_*l*_ ≤ π/2. If Θ(*V*_1_, *V*_2_) = [θ_1_, θ_2_, …, θ_*l*_] then *d*_*g*_(*V*_1_, *V*_2_) = ||Θ(*V*_1_, *V*_2_)||_2_ is known as the geodesic norm between *V*_1_ and *V*_2_. If sinΘ(*V*_1_, *V*_2_) = [sinθ_1_, sinθ_2_, …, sinθ_*l*_] then *d*_*c*_(*V*_1_, *V*_2_) = ||sinΘ(*V*_1_, *V*_2_)||_2_ is known as the chordal norm between *V*_1_ and *V*_2_.

We can generalize to the setting where *V*_1_ and *V*_2_ have potentially differing dimensions and we can modify the measure of the length of the principal angle vector between these subspaces. We will rename *V*_1_ as *V* and *V*_2_ as *W* to emphasize this flexibility in dimensional differences. Let *V, W* be subspaces of ℝ^*n*^ of dimensions *l* and *k* and let *m* = min(*l, k*). Let *c* be a positive integer less than or equal to *m* and define Θ_*c*_(*V, W*) = [θ_1_, θ_2_, …, θ_*c*_] and sinΘ_*c*_(*V, W*) = [sinθ_1_, sinθ_2_, …, sinθ_*c*_]. Given a vector norm, ||·||_α_ on ℝ^*c*^, we can measure the “size” of the vector Θ_*c*_(*V, W*) or of sinΘ_*c*_(*V, W*). We claim that in either case, this norm gives a measure of closeness between a point *V*∈*GR*(*l, n*) and the Schubert variety Ω_*c, k, l*_(*W*) by defining *d*(*V*, Ω_*c, k, l*_(*W*)) = ||Θ_*c*_(*V, W*)||_α_ or by defining *d*(*V*, Ω_*c, k, l*_(*W*)) = ||sinΘ_*c*_(*V, W*)||_α_. To see that this is a measure of closeness, note that θ_1_, …, θ_*c*_ are the *c* smallest principal angles between the subspaces. They correspond to the *c* smallest possible principal angles between *c*-dimensional subspaces of *V* and *c*-dimensional subspaces of *W*.

If we now pick a norm ||·||_β_ on ℝ^*r*^, once we have chosen a norm for measuring the size of Θ_*c*_(*V, W*) or of sinΘ_*c*_(*V, W*), we can measure a Schubert variety's fit to a collection of *l*-dimensional subspaces *D* = {*V*_1_, …, *V*_*r*_} of *R*^*n*^ by


$$\begin{array}{l} \;FIT(D,\Omega_{c,k,l}\left(W\right))=\\ ||d\left(V_{1},\Omega_{c,k,l}\left(W\right)\right),d\left(V_{2},\Omega_{c,k,l}\left(W\right)\right),\ldots ,d\left(V_{r},\Omega_{c,k,l}\left(W\right)\right)|{|}_{\beta } \end{array}$$

and we can define the Schubert variety of best fit to*D* as $BEST\left(D,\Omega_{c,k,l}\right)=\Omega_{c,k,l}\left(W^{*}\right)$ where


$$\begin{array}{l} W^{*}=arg\;min_{W\in GR\left(k,n\right)}FIT\left(D,\Omega_{c,k,l}\left(W\right)\right) \end{array}$$

For programming advantages, in the next section an optimization problem is described in terms of maximizing the norm of cosΘ_*c*_(*V, W*) = [cosθ_1_, cosθ_2_, …, cosθ_*c*_] instead of minimizing the norm of sinΘ_*c*_(*V, W*). The goal of the optimization problem is to find *BEST*(*D*, Ω_*c, k, l*_).

## 4 Optimization problem for SVBF

Consider a set of matrices ${\left\{X_{i}\right\}}_{i=1}^{r}$ with each $X_{i}\in {\mathbb{R}}^{n\times l}$ having orthonormal columns. Let *K*∈ℝ^*n*×*k*^ be an unknown matrix with orthonormal columns. Let ℛ(*X*_*i*_) denote the column space of *X*_*i*_. Define θ_*ij*_ to be the *j*th smallest principal angle between ℛ(*X*_*i*_) and ℛ(*K*).

*Problem Statement: Given the set* ${\left\{X_{i}\right\}}_{i=1}^{r}$ *and an integer* *c* *with* 1 ≤ *c* ≤ *min*(*l, k*)*, find* *K* *that comes as close as possible to satisfying* *dim*(ℛ(*X*_*i*_)∩ℛ(*K*))≥*c* *for each of the subspaces* ℛ(*X*_*i*_).

One approach is to find a matrix K^*^∈ℝ^*n*×*k*^ such that


(1) $$\begin{array}{l} K^{*}=\arg\underset{K\in {\mathbb{R}}^{n\times l}}{\max}\frac{1}{rc}\overset{r}{\underset{i=1}{\sum }}\overset{c}{\underset{j=1}{\sum }}\cos^{2}\left(\theta_{ij}\right) \end{array}$$

subject to *K*^*T*^*K* = *I* and 1 ≤ *c* ≤ min{*l, k*}. We have normalized by the factor 1/*rc* so that the optimal value of the solution will be one. Note that for *c* = min{*l, k*} this is the flag mean (Marrinan et al., 2015).

Alternatively, we can solve


(2) $$\begin{array}{l} K^{*}=\arg\underset{K\in {\mathbb{R}}^{n\times k}}{\max}\frac{1}{rc}\overset{r}{\underset{i=1}{\sum }}\overset{c}{\underset{j=1}{\sum }}\cos\left(\theta_{ij}\right) \end{array}$$

subject to *K*^*T*^*K* = *I* and 1 ≤ *c* ≤ min{*l, k*}. Note that for *c* = min{*l, k*} this is the flag median (Mankovich et al., 2022).

### 4.1 SVBF optimization problem formulation

For simplicity, in this paper we will focus on the case *c* = 1, i.e., we are looking for the column space of each *X*_*i*_ to be close to intersecting the column space of *K* in at least one dimension.

Suppose we are given a set of matrices ${\left\{X_{i}\right\}}_{i=1}^{r}$, each $X_{i}\in {\mathbb{R}}^{n\times l}$ satisfying $X_{i}^{T}X_{i}=I_{l}$. We would like to find a matrix *K* whose column space intersects the column space of each *X*_*i*_ in at least a one dimensional space. This is achieved if and only if the first principle angle between the column space of *K* and the column space of *X*_*i*_ is equal to zero for each *i*. Let θ_1_(*K, X*_*i*_) denote the first principle angle between the column space of *K* and the column space of *X*_*i*_. In the optimization below we seek to find a matrix *K* that maximizes the function $\overset{r}{\underset{i=1}{\sum }}\cos^{2}\left(\theta_{1}\left(K,X_{i}\right)\right)$. This amounts to finding a a matrix K^*^∈ℝ^*n*×*k*^ such that


(3) $$\begin{array}{l} K^{*}=\arg\underset{b_{i},K}{\max}\overset{r}{\underset{i=1}{\sum }}b_{i}^{T}K^{T}X_{i}X_{i}^{T}Kb_{i} \end{array}$$

subject to $b_{i}\in {\mathbb{R}}^{k\times 1}$, ||*b*_*i*_|| = 1, *K*∈ℝ^*n*×*k*^, and $K^{T}K=I_{k}$.

In the second optimization problem, given below, we seek to find a matrix *K* that maximizes the function $\overset{r}{\underset{i=1}{\sum }}\cos\left(\theta_{1}\left(K,X_{i}\right)\right)$. This amounts to finding a matrix K^*^∈ℝ^*n*×*k*^ such that


(4) $$\begin{array}{l} K^{*}=\arg\underset{a_{i},b_{i},K}{\max}\overset{r}{\underset{i=1}{\sum }}a_{i}^{T}X_{i}^{T}Kb_{i} \end{array}$$

subject to $a_{i}\in {\mathbb{R}}^{l\times 1}$, ||*a*|| = 1, $b_{i}\in {\mathbb{R}}^{k\times 1}$, ||*b*_*i*_|| = 1, *K*∈ℝ^*n*×*k*^, and $K^{T}K=I_{k}$.

### 4.2 SVBF optimization problem implementation with PyTorch

All of the experiments in this paper are conducted for the special case where *c* = 1. This case, along with the relative simplicity of the dataset that we used, allows us to directly initialize the problem with the PyTorch class. The unknown matrix *K* and vectors {*b*_*i*_} in this case are initialized in the PyTorch class as two sets of parameters: {*K*_*ij*_}, 1 ≤ *i* ≤ *n*, 1 ≤ *j* ≤ *k*, and {*b*_*ij*_}, 1 ≤ *i* ≤ *r*, 1 ≤ *j* ≤ *k*. The Lagrangian, associated with the problem given by Equation (3), is considered via the Adam optimizer and provides a path to the approximate solution:


(5) $$\begin{array}{l} \;Loss_{K}\left(K,b\right)=\\ -\sum_{i=1}^{r}b_{i}^{T}K^{T}X_{i}X_{i}^{T}Kb_{i}+\lambda_{0}\left(K^{T}K-I_{k}\right)+\sum_{i=1}^{r}\lambda_{i}\left(\|b_{i}\|-1\right) \end{array}$$

Let's focus on the first part of this expression as the part containing a majority of the complexity. In our design, for each sample it is calculated in three steps:

1.
$L_{i}^{1}=K^{T}X_{i}$

2.
$L_{i}^{2}=L_{i}^{1}L_{i}^{1T}$

3.
$L_{i}^{3}=b_{i}^{T}L_{i}^{2}b_{i}$

Based on this chain of matrix multiplications and assuming that *n*≫*k* we can calculate the time complexity of the forward pass as 𝒪(2*k*(*nl*+*kl*+2*k*)) = 𝒪(*nkl*). The backward pass complexity, calculated based on the chain of multiplications of respective Jacobian matrices, is also equal to 𝒪(*nkl*). Hence, along with complexity of the Adam optimizer, equal to 𝒪(*nk*), the overall complexity of one iteration is equal to 𝒪(*nkl*) and the complexity of the entire optimization process is equal to 𝒪(*tnklr*), where *t* is the number of iterations and *r* is a number of *l*-dimensional inputs. Note that it is effectively equal to the complexity of the training of one fully connected layer with *k* nodes at the output given that it is trained on *l*×*r* *n*-dimensional vectors with the same number of iterations *t*.

The given approximations of the complexity are derived following the traditional approach that was common before the era of GPU's. The GPU's benefit from parallelizing an immense number of simple operations and are capable of accelerating matrix multiplications by orders of magnitude. We run all of our experiment on V-100 Tesla GPU's, hence to provide a better understanding of performance in actual experiments we present a computational profiling in Table 1. The profiling was conducted for 100 randomly generated inputs. The dimensionalities of inputs vary as *n*∈{8^2^, 8^3^, 8^4^, 8^5^}, *l*∈{1, 4^1^, 4^2^, 4^3^}, while the dimensionality of *K* varies as *k*∈{8 × 1, 8 × 4^1^, 8 × 4^2^, 8 × 4^3^}. Given that SVBF optimization can also be run as a training with batches for larger number of samples, this range of parameters supposedly covers several of the possible cases that one may encounter in real experiments.

**Table 1:** Tables of processing times for one iteration of optimization loop in milliseconds. The number of samples is fixed to 100, while the dimensionatity of inputs *l*, dimensionalilty *k* of K, and dimensionality *n* of ambient space vary.

***l*\*n***	**8^2^**	**8^3^**	**8^4^**	**8^5^**
1	1.18	1.23	1.58	2.77
4^1^	1.27	1.70	5.00	49.31
4^2^	1.26	1.69	5.00	45.27
4^3^	1.30	1.82	5.20	48.80
*l*\*n*	8^2^	8^3^	8^4^	8^5^
1	1.30	1.49	2.45	1
4^1^	1.47	1.97	5.09	28.18
4^2^	1.47	1.98	5.18	30.53
4^3^	1.54	2.13	5.87	52.20
*l*\*n*	8^2^	8^3^	8^4^	8^5^
1	1.54	2.13	8.62	160.36
4^1^	1.86	2.72	10.43	191.06
4^2^	1.87	2.76	10.91	192.08
4^3^	2.01	3.01	11.99	204.16
*l*\*n*	8^2^	8^3^	8^4^	8^5^
1	4.09	6.76	73.90	491.70
4^1^	5.17	7.72	77.40	506.52
4^2^	5.49	7.97	80.87	518.80
4^3^	6.58	9.96	89.52	616.49

*k* = 8 × 1. *k* = 8 × 4^1^. *k* = 8 × 4^2^. *k* = 8 × 4^3^.

In fact, instead of including the unit length condition for each of the *b*_*i*_'s in the cost function, we use the normalizing transformations of *b*_*i*_'s suggested in Kirby and Miranda (1996), which doesn't introduce much complexity and, at this point, has already been implemented in PyTorch as a built-in routine. The resulting loss function is as follows:


(6) $$\begin{array}{l} Loss_{K}\left(K,b\right)=-\overset{r}{\underset{i=1}{\sum }}b_{i}^{T}K^{T}X_{i}X_{i}^{T}Kb_{i}+\lambda_{0}\left(K^{T}K-I_{k}\right) \end{array}$$

where ||*b*_*i*_|| = 1∀*i*∈{1, *r*} by construction of the PyTorch class. As it was mentioned before this function is minimized with Adam optimizer and the number of iterations is equal to 40,000. The following is a list of some other details important for reproduction of results:

- λ_0_ = 10,000
- Initialization of parameters K and {*b*_*i*_}:
  element-wise random numbers from a uniform distribution on the interval [0,1]
- Adam optimizer settings:
  |lr = 0.001, betas = (0.9,0.999), eps = 1e-08, weight_decay = 0, amsgrad = False|
- Python version: 3.6.8
- torch version: 1.10.1
- cuda version: 11.0

All these settings are preserved exactly the same across all experiments including SVBF problem solving. For the faster processing in benchmarking experiments we also leverage Ray 2.0.0 python package to run several experiments in parallel.

### 4.3 Illustrative example

**Figure 2:**
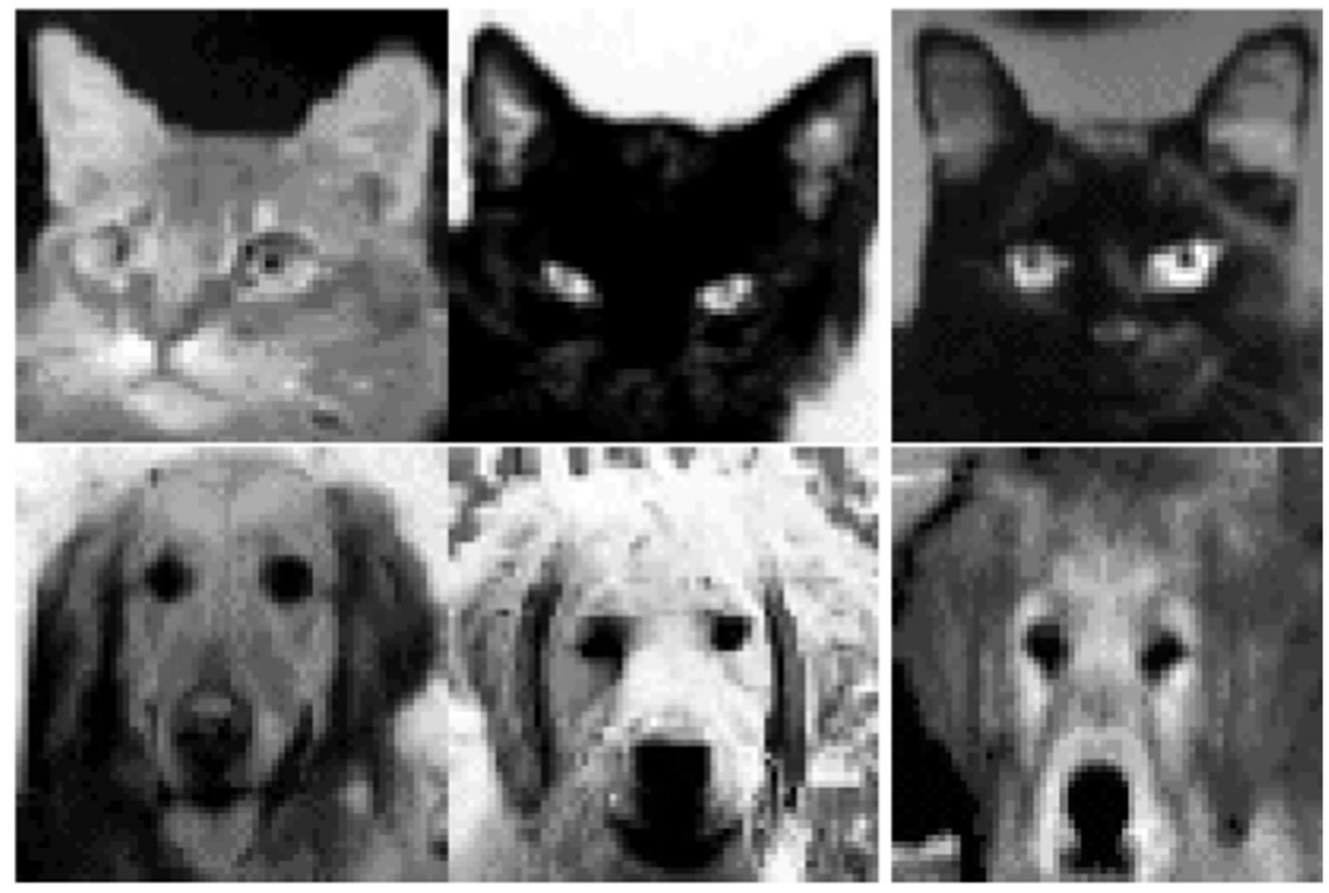
Images from the Cat and Dog dataset. The **(top)** row corresponds to the data in a single cat sample with three elements. Similarly, the **(bottom)** row corresponds to a dog sample consisting of three elements.

In this paper we now present results from the given implementations of the SVBF algorithm. Given that our objective is a comparative analysis of these algorithms, we focus on a modestly sized Cat and Dog dataset consisting of 99 images of cats and 99 images of dogs. All the images are 64 × 64 greyscale images; you can see some of the representatives of each class in Figure 2. We preprocess the data by flattening each image into a vector of length *n* = 4, 096 and scaling the entries into the range from −1 to 1. The SVBF algorithm operates over sets of subspaces, therefore we split the data into equally sized collections of *l* vectors and extract an orthonormal basis for each set. The resulting orthonormal bases are used as the inputs. In other words, the inputs, or samples, consist of tall orthonormal matrices of dimension ℝ^*n*×*q*^ where *q* = *l* for training data and *q* = *m* for test data. We chose to allow for *l* and *m* to be distinct so that we may consider the cases when dimensionality of samples in training and test data might differ. Importantly, no cat or dog vector is used in more than one sample. Further, we never mix classes within one sample, samples consist of sets of only dog vectors or only cat vectors. For example, the notations $\left\{X_{i}^{train}\right\}$, *l* = 3 describes the set of three-dimensional bases of mono-class sets of scaled image-vectors sampled from the training data.

**Figure 3:**
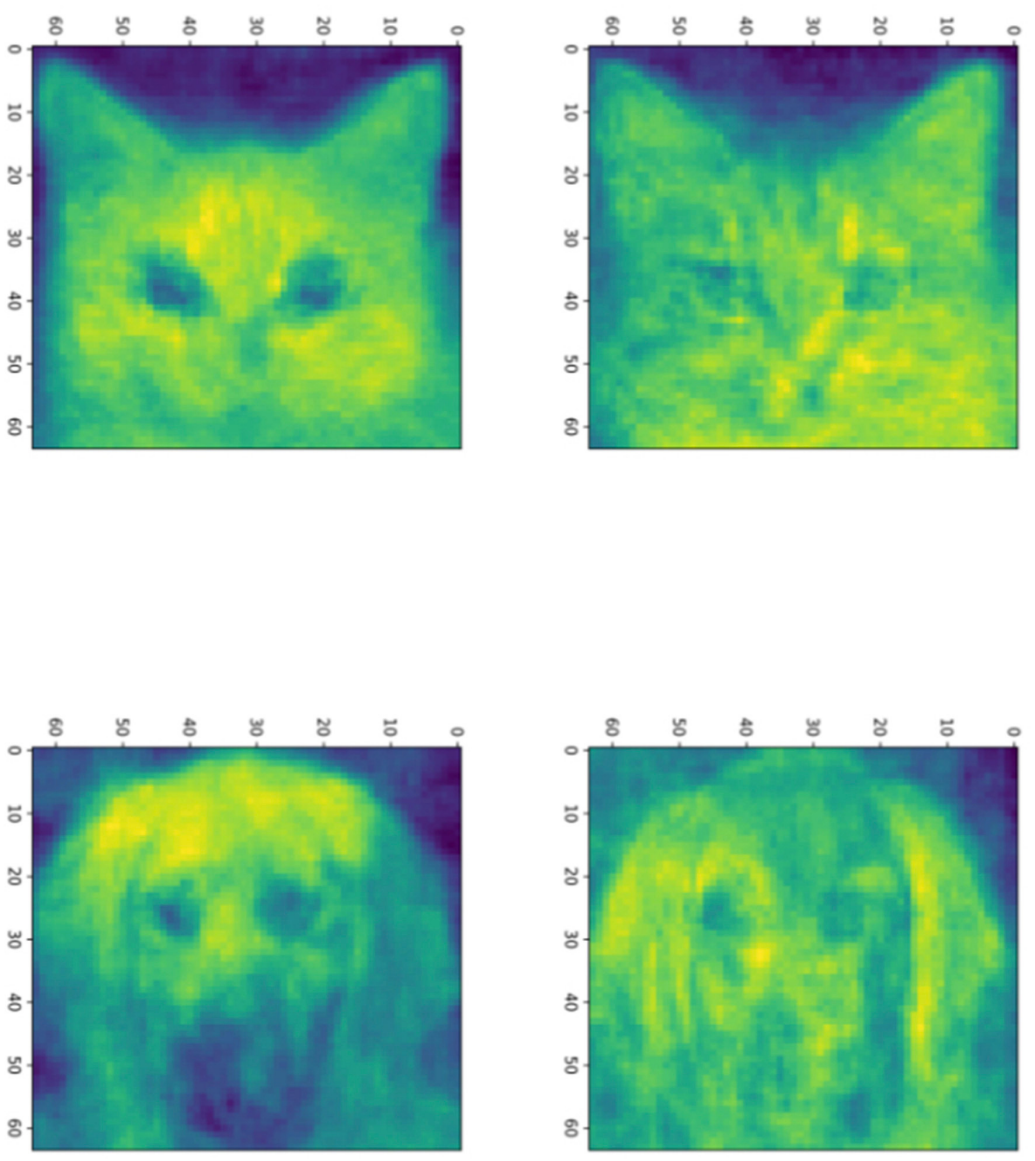
Solutions to the Schubert Variety of best fit problem for *a* = 1. The **(left)** column consists of solutions to Equation (3) while the **(right)** column consists of solutions to Equation (4). In each case there are three images in each sample. The **top** and **bottom** rows use cat and dog samples, respectively.

In Figure 3, we show sample solutions of Schubert Varieties of Best fit. In each case the matrix *K* to be determined is chosen to have one column and these solutions are displayed for cats and dogs individually. It is interesting to compare the solutions to Equation (3) and Equation (4). We see that for *cos*(θ) the Schubert variety matrices *K* show higher resolution detail while for cos^2^(θ) the features are larger scale. This example underscores the potential significance in the selection of distance measure in computing SVBFs. For all these examples we select three images in each sample.

### 4.4 Optimal dimension of K

**Figure 4:**
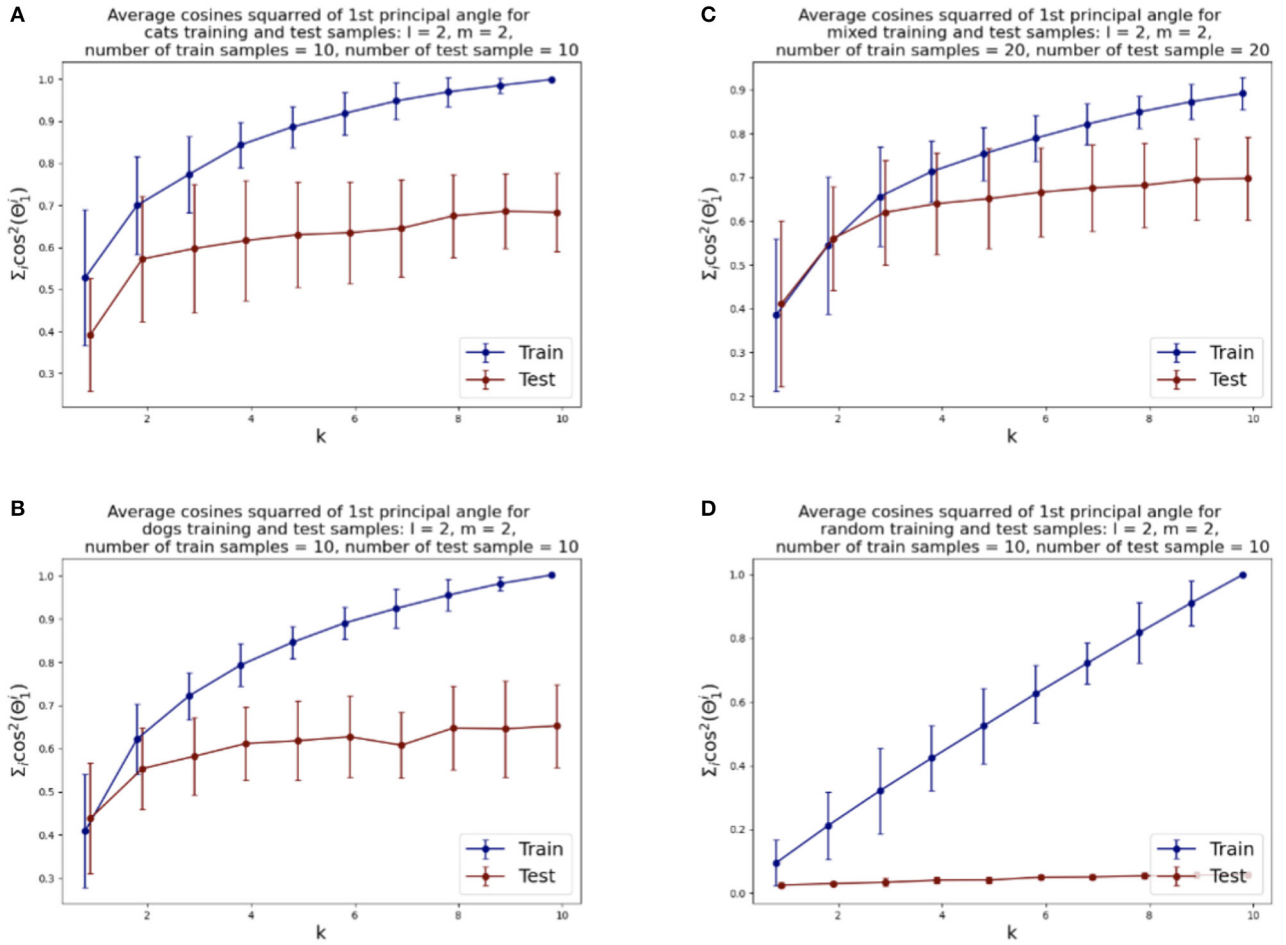
Average squared-cosines of the 1st principle angle for training and test data sampled from different classes, from both classes and sampled from the uniform distribution on the Grassmann manifold: **(A)** cat class, **(B)** dog class, **(C)** cats and dogs classes combined, **(D)** random samples.

We illustrate the relative rates of conve rgence of the SVBF optimization problems in Figure 4. Figure 4A shows the value of the objective function for the optimization problem given by Equation (3), i.e., the average sum of the squared cosines of the 1st principal angles for 10 two-dimensional subsets of cats sampled from both training and test datasets versus the dimension *k* of the solution subspace *K*. We can see that the objective function for test samples effectively flattens out after *k*=2. Similar behavior can be observed for the dog dataset, presented in Figure 4B, except the flattening is smoother and starts approximately at *k* =4. The results for the combined datasets, presented in Figure 4C, show that the flattening also starts at around *k* = 4. These plots lead us to several observations. Firstly, the flattening of the curves itself suggests that more columns in *K* do not lead to improvements in the solution. Thus, the set of points {*X*_*i*_} has an intrinsic subspace dimension as captured by *K*. We see that the dimension of this subspace is smaller than the direct sum of the dimensions of the class-specific subspaces. This does not come as too big a surprise when one considers the correlation between the images. Finally, the Figure 4D illustrates how samples, randomly selected from the uniform distribution (with respect to Haar measure) on a Grassmann manifold, do not possess the same structure and the number of columns required in *K* does not converge. Experiments with other dimensions of samples have also been conducted, and they align with the results reported here. Other approaches to dimension optimization are also possible with the SVBF method, e.g., task-specific optimizations that will be considered in Section 5.

## 5 SVBF as an abstract node

Here, we explore the application of the SVBF as a computational unit in a feed-forward neural network. We shall see that Schubert varieties of best fit provide a natural transformation of the data into the coordinates of the learned Schubert variety of best fit *K*.

### 5.1 Algorithm I

Given a solution *K* and a data sample *X*_*i*_ it is natural to consider the change of coordinates of the sample that can be implemented in the neural network, i.e.,


$$Y_{i}=K^{T}X_{i}$$

The design of the classification experiment for this case is presented in Figure 5.

**Figure 5:**
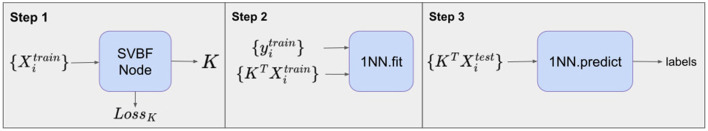
Workflow of Cat and Dog classification experiment using Algorithm I.

Step 1 involves training the SVBF Node, the outcome of which is the representative *K* of the SVBF. This *K* is then used in Step 2 to compute the change of coordinates to produce Y. This is done using training data. Further, in Step 2 the 1-nearest neighbor (1NN) classification is implemented using the scikit-learn Python package. Step 3 involves the application of 1NN to the test data and the results are shown in Figure 6. Figure 6A illustrates the average for 10 different samplings of a 1NN-classifier applied to the {*Y*_*i*_} embeddings of Cat and Dog data. In each sampling, the data is split randomly into train and test datasets with proportions 0.78 and 0.22, respectively. Benchmarking is performed for different dimensions *l* = *m* = 1, …, 5 of samples in training and tests subsets, and different dimensions *k* = 1, …, 10 for *K*. The accuracy grows with the dimension of the samples, but interestingly starts decreasing for *l*≥3. At the same time, increasing the dimension of *K* also significantly increases the accuracy up to dimension 3 and only slightly effects the accuracy above that level. This low accuracy is a result of using the Frobenius norm to compute distances between matrices. Later we will see that using subspace distances in general produces superior results. For a better understanding of the benefits of the SVBF method, we provide side by side the results for classification based on PCA-embeddings as well (Figure 6B). In this first experiment, the performance of the classification method based on learning *K* is slightly better than the one based on learning a representative subspace based on PCA-analysis.

**Figure 6:**
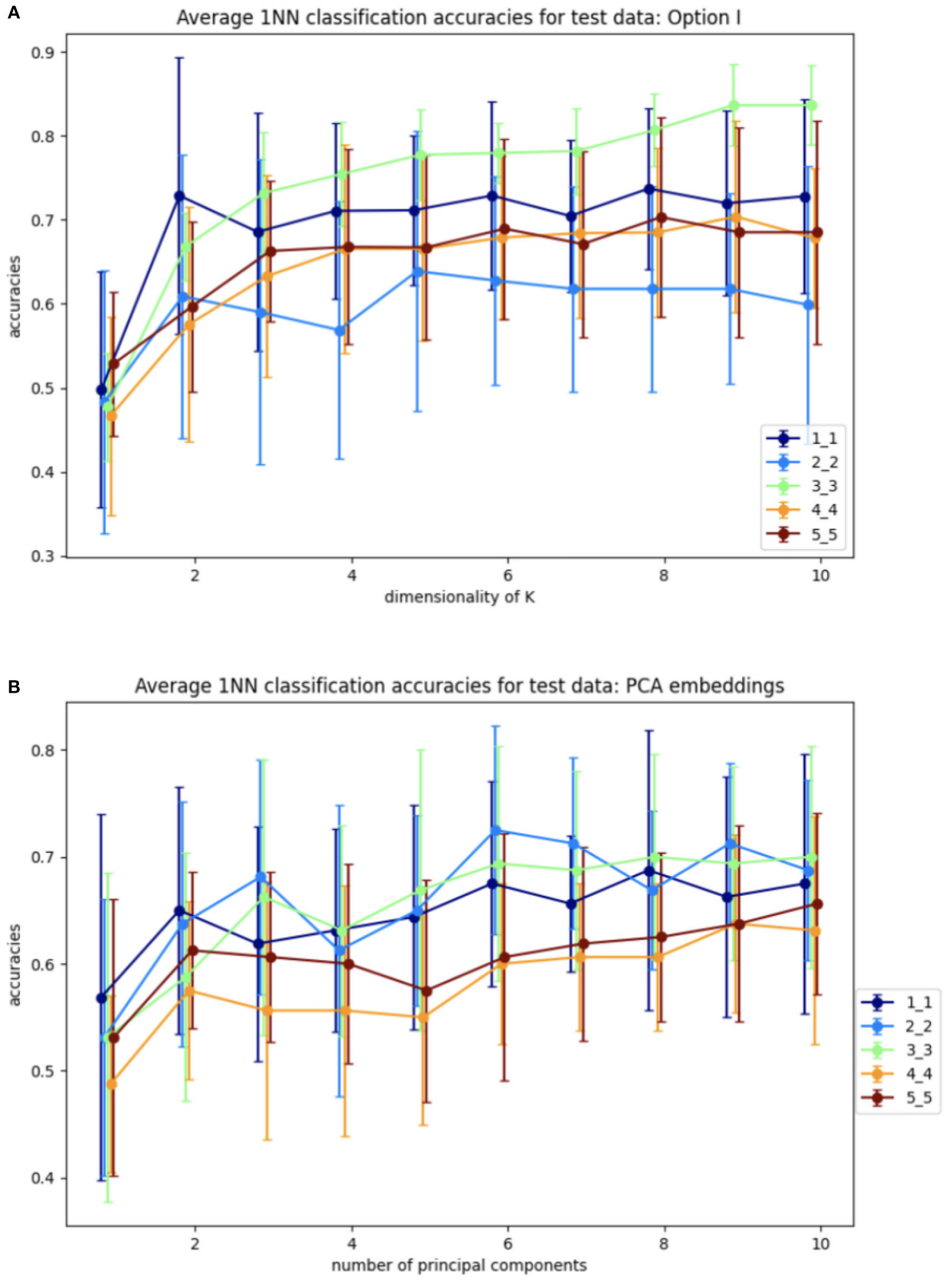
Average accuracies with error bars for 1NN-classification of test data for Y embeddings from Algorithm I and for PCA embeddings. **(A)** Y embeddings, plots are labeled as *l*_*m* depending on the dimensions of training samples *l* and dimensions of test samples *m*. **(B)** PCA embeddings, plots are labeled as *m*_*m* depending on dimensions of test samples *m*.

### 5.2 Algorithm II

Another possible approach is to learn a representative *K* for each class of data. In our example, in Step 1, we propose to learn *K*_1_ as a solution to Equation (3) for the cat class, and *K*_2_ as a solution to Equation (3) for the dog class. Now these matrices *K*_1_, *K*_2_ can be used to classify the data. To assign a class to an unknown sample *X*_*i*_ in Step 2, we compute the smallest principle angle between *X*_*i*_ and each of *K*_1_ and *K*_2_. The smallest of these two angles provides the classification. The diagram for such an experiment with the Cat and Dog dataset is presented in Figure 7. Labeling of the test data is performed by finding the nearest, in terms of smallest principle angle to *K*, for each sample.

**Figure 7:**
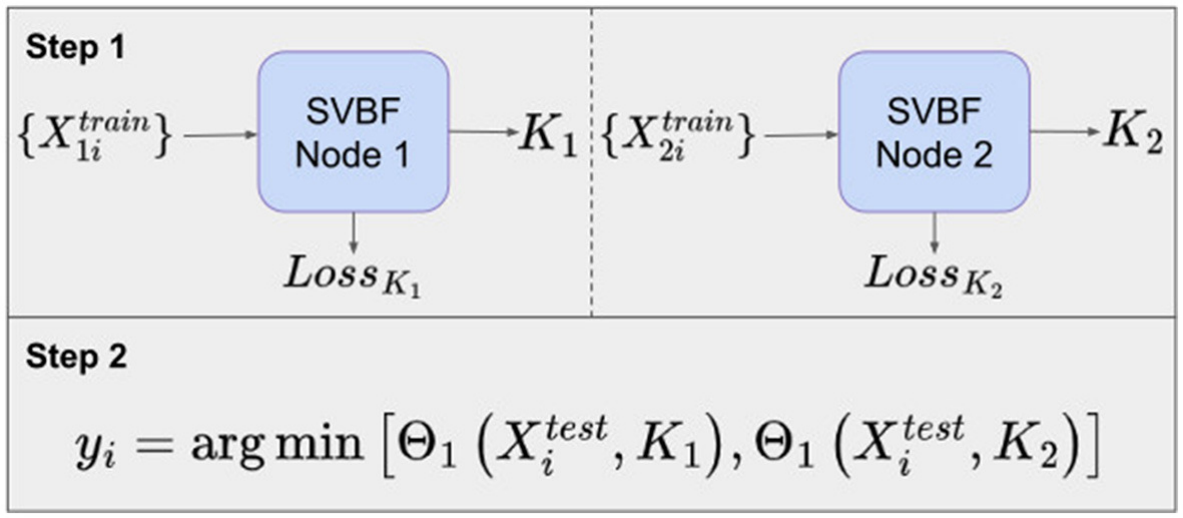
Workflow of Cat and Dog classification experiment using Algorithm II.

Figure 8A shows the average classification accuracy across 10 different samplings of the test data. Again, this procedure is implemented for a range of *l, m*, and *k*. The data was split into training and test sets with the same ratio as in the Algorithm I experiments.

**Figure 8:**
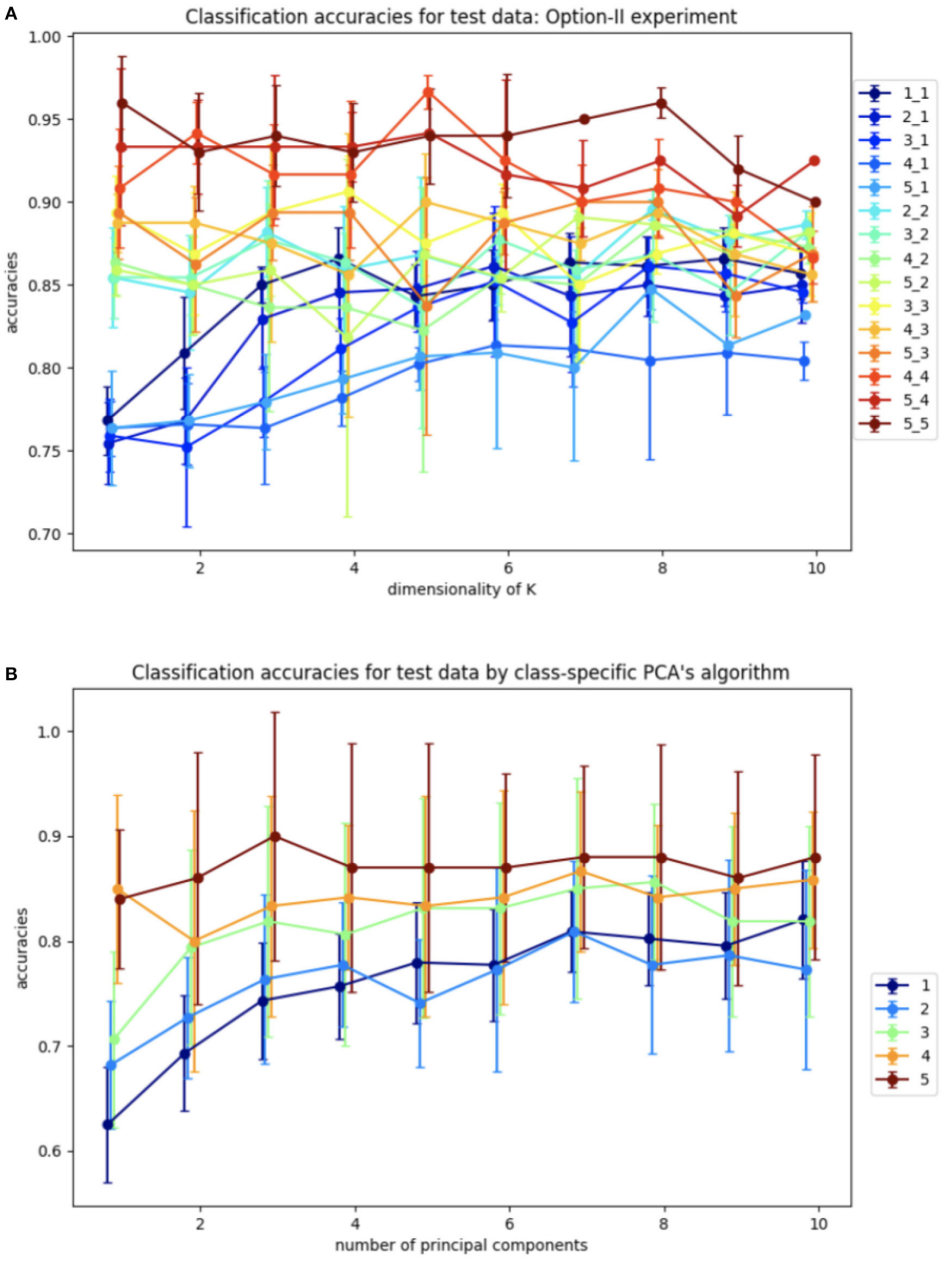
Average accuracies of classification of test set by Algorithm II design and design based on the closest class-specific PCA subspaces: **(A)** Algorithm II design, plots are labeled as *l*_*m* depending on the dimensions of training samples *l* and dimensions of test samples *m*. **(B)** Closest PCA design, plots are labeled as *m* depending on dimensions of test samples *m*.

The benchmarking plots indicate a significantly more accurate classification versus Algorithm I for the comparable cases. Due to the higher overall accuracy of this design, we also investigated it for different dimensions of training and test samples, which can be useful for a wide variety of datasets, specifically when test samples cannot be grouped by labels. As before, increasing the dimension *k* of *K* leads to higher accuracies for all cases. However, in contrast to Algorithm I, now the accuracy increases monotonically with the dimensions *l, m*.

For further comparative analysis, we also calculate PCA embeddings for each class and label test samples, based on the nearest, in terms of the smallest principle angle subspace captured by PCA-analysis. We repeat this experiment 10 times for different samplings similar to the Algorithm I experiment. The results of this experiment are shown in Figure 8B. The lower accuracies for the class-specific PCA experiment indicate the SVBF optimization problem is capturing additional useful information that is helpful for classification.

### 5.3 Algorithm III

Note that the element $\hat{k}\in ℛ\left(K\right)$ given by


$${\hat{k}}_{i}=Kb_{i}$$

is the vector in ℛ(*K*) that is closest to the span of *X*_*i*_. There is a natural association between *X*_*i*_ and *b*_*i*_ and this can be exploited for classification. Hence, a consequence of Equation (3) is that once we approximate *K* we can use *b*_*i*_, the coordinates of the closest to *X*_*i*_ vector in *K*, as a proxy for *X*_*i*_ in the classification. One of the possible experiments exploiting this option is classification with neural networks outlined in Figure 9.

**Figure 9:**
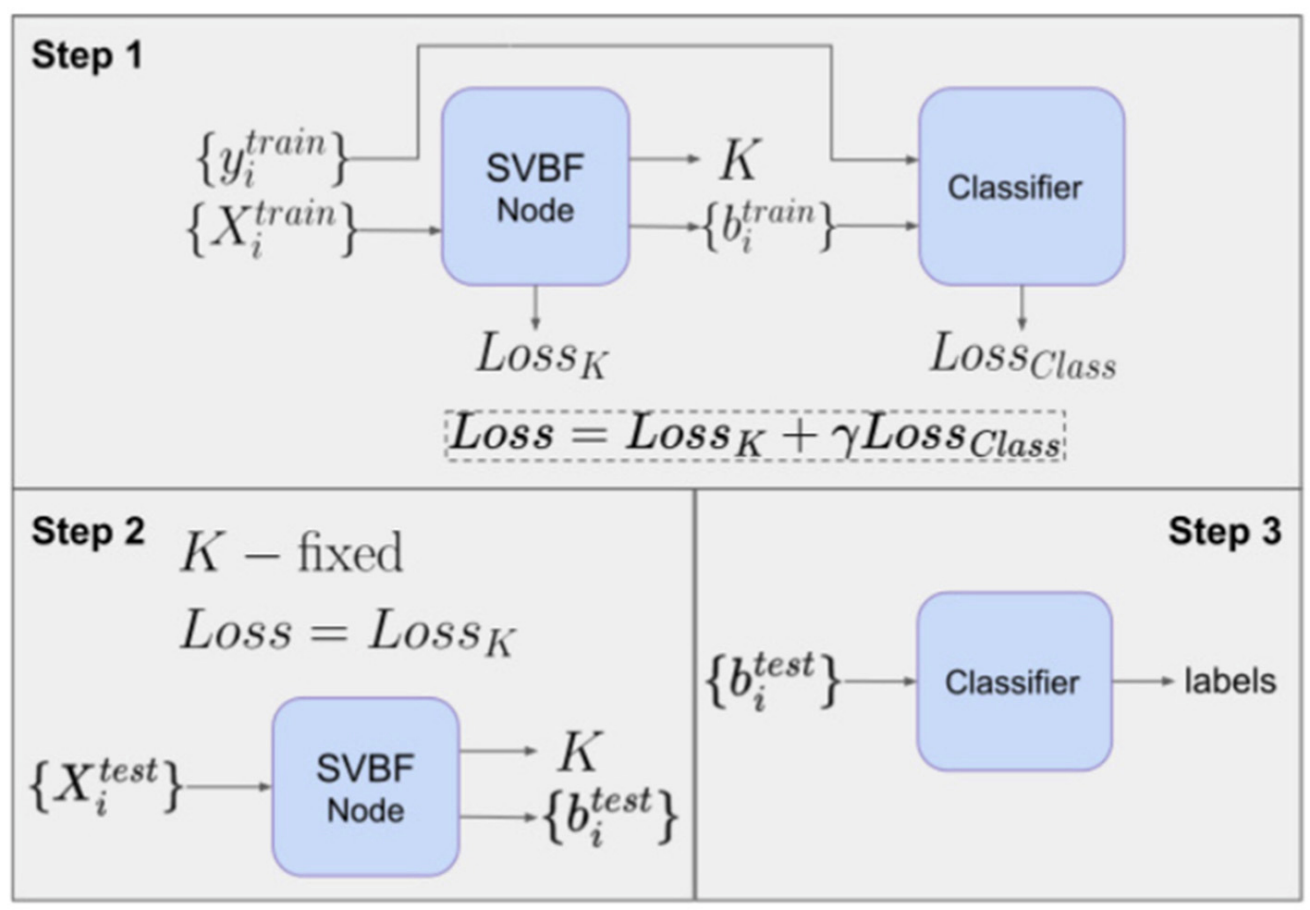
Workflow of Cat and Dog classification experiment using Algorithm III.

This experiment also illustrates how SVBF nodes can be stacked into a larger network and trained simultaneously with other parts of the network. In this case, we attach a classifier that takes outputs *b*_*i*_ of SVBF nodes as inputs, learns centroids in a training process and generates soft labels at the inference step. In Step 1, we optimize *K* and *b*_*i*_ independently at each iteration. In other words, *K* is not adjusted by any contribution to the gradient from the classification error. Generally, the outline given in Figure 9 does not require detaching the classifier's error back-propagation from that of an SVBF node. However, detaching the training of *K* and *b*_*i*_, as we have done here, seems to improve performance without loss of the benefits of parallelization. The loss function for Step 1 is defined as a weighted sum of three loss functions:


(7) $$\begin{array}{l} \begin{array}{l} Loss_{\theta }=-\sum_{i=1}^{M}b_{i}^{T}K^{T}X_{i}X_{i}^{T}Kb_{i}\\ Loss_{orth}=K^{T}K-I_{k\times k}\\ Loss_{Class}=-\sum_{j=1}^{k}y_{ij}\log\left({\text{logit}}_{ij}\right)\\ Loss=\alpha Loss_{\theta }+\beta Loss_{orth}+\gamma Loss_{Class} \end{array} \end{array}$$

where ${\text{logit}}_{i}=Softmax\left({\left(b_{i}^{T}W\right)}^{2},T\right)$ with *W* learnable centroids, and the hyperparameter *T*. The output of Step 1 is the matrix *K*, set $\left\{b_{i}^{train}\right\}$ and the classifier *W*. Now, given *K, W*, the prediction of the labels is accomplished in Steps 2 and 3.

In Step 2, we initialize the SVBF node with the *K* computed in Step 1 to determine the coordinates $b_{i}^{test}$, associated with the test samples $X_{i}^{test}$, using the loss function *Loss*_*K*_ similar to Step 1, but without the classification component and with *K* fixed. In Step 3, we use the classifier *W* trained in Step 1 to map the $b_{i}^{test}$ to their class label.

This hybrid network was also tested with the Cat and Dog dataset following exactly the same preprocessing pipeline as in the Algorithm I and Algorithm II experiments. Figure 10 illustrates trained parameters for such a network for the entire Cat and Dog dataset with *l* = 2 and *k*=3. As we now describe, this picture captures many interesting features of the method. Figure 10A depicts the three columns of *K*. Interestingly one column is a dog, and one is clearly a cat! But all columns seem to capture salient features in the data. In Figure 10B, we show the directions *Kb*_*i*_ for three cat samples and three dog samples, where we recall that each sample is a 4, 096 × 2 matrix. So each of these six figures is a direction in the span of *K* that is closest to the data training sample. In Figure 10C, we show the set of all features in terms of *b*_*i*_ for cats (blue) and dogs (red). We note that their clear separation is a reflection of the fact that this method captures information capable of discriminating between cats and dogs.

**Figure 10:**
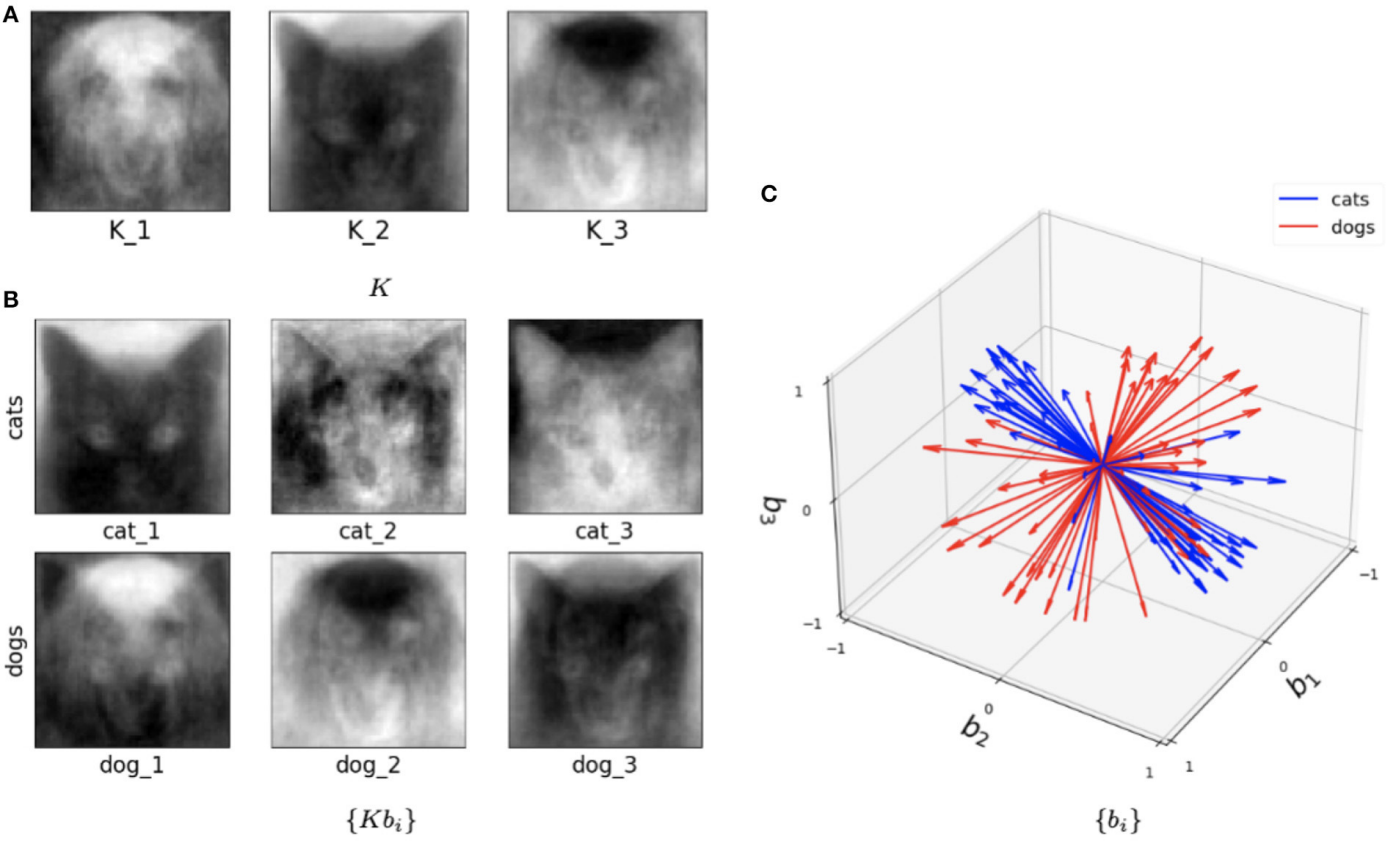
Visualization of the outputs of the trained SVBF node and generated embeddings for the entire Cat and Dog dataset, *k*=3, and *l*=2. **(A)** Learned basis *K*, **(B)** some samples reconstructed in ambient space from embeddings, **(C)** learned embeddings.

In Figure 11, you can find the classification accuracy for test data for different dimensions of training and tests samples as well as different dimensions for *K*. It is very interesting that the accuracy of the classification problem levels off at 4 dimensions, which is consistent with the optimal size *k* = 4 of *K* as suggested in Figure 4. Hence we view *k* = 4 as the apparent *working* dimension for *K*. We see the accuracy increases monotonically with the dimension of the samples. Importantly, the results in which the samples have dimensions >5, which we are not reporting here, support this observation. However, given our limited data we were not able to pursue this behavior further.

**Figure 11:**
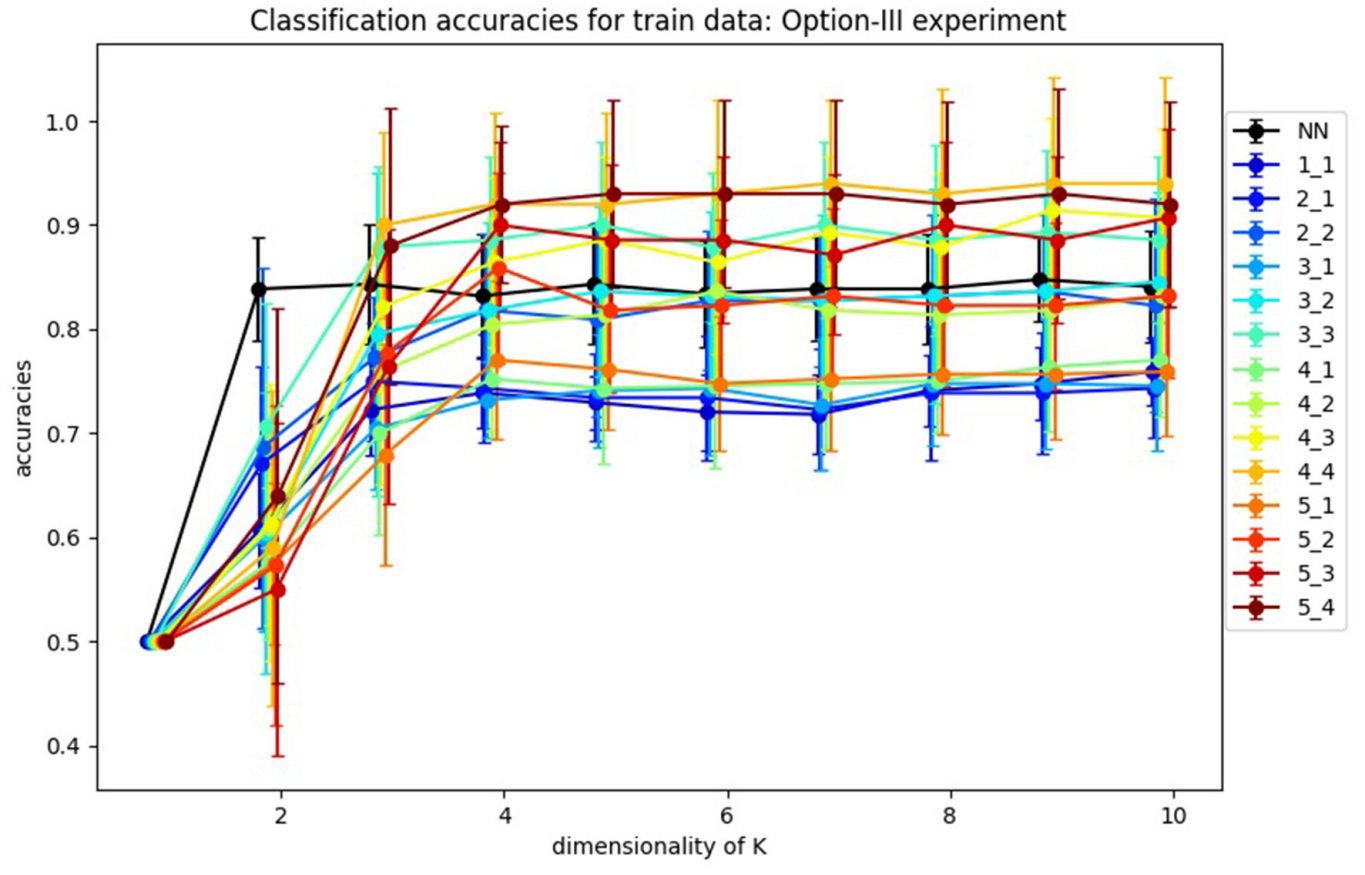
Average accuracies of classification of test data by Algorithm III design. Accuracies for one-layer NN design are labeled as “NN,” and accuracies for SVBF-Node design as “*l*_*m*,” where *l* is dimensionality of train samples and *m* is dimensionality of test samples.

In the same Figure 11, we also report the average accuracies for the classification of *l*_2_-normalized vectors used as inputs. In this design, instead of an SVBF-Node, we use one fully connected layer with *k* nodes at the output and with the hyperbolic tangent as the activation function (all other settings are kept the same). In Section 4.2, it was shown that this network has the same computational complexity as the SVFB-Node. Along with the higher resulting average accuracies for some cases this method also has an advantage of ~10 times faster convergence (in terms of the required number of iterations). On the other hand, we can see that for dimensionalities of test samples ≥3 the SVBF method is more accurate. It's important to note here that benchmarking against the regular networks processing vector inputs wasn't within the focus of this paper. Our prime goal was to show that in the suggested framework a big part of the progress made with the regular neural networks during the last decades can be directly leveraged in the case of sets of datasets.

## 6 Conclusions and future work

In this paper we proposed a geometric approach for the analysis of sets of datasets using the idea of a Schubert Variety of Best Fit. We formulated two optimization problems and compared them on a two class data set comprised of sets of cats and dogs. We proposed three distinct algorithms for data classification and explored and benchmarked these using the same dataset and preprocessing pipeline. Algorithm I uses a single solution *K* to characterize the data and a Frobenius norm to measure distances. Overall, this algorithm performed poorly when compare to Algorithms II and III which generated promising results. Algorithm II, based on learning two SVBFs, provides the most accurate classifications. This algorithm explicitly builds a model for the cats and dogs through their respective SVBFs and appears to be more capable of addressing complex structures of data, including classes with higher within-class variance. Algorithm III introduces the idea of using auxiliary features to perform data classification, and is based on a single model *K*.

All three algorithms suggest that there is a best dimension for the representative subspace and that exceeding this leads to no improvement in classification accuracy. Hence the SVBF approach appears to provide a measure of complexity of a set of data sets through this *working* dimension. Interestingly, Algorithm III provides a very clear signal for this optimal dimension through a classification criterion. Algorithm III illustrates how the SVBF node can be used as an abstract unit of computation in a neural network. This enables researchers to process sets of datasets in the same spirit as sets of vectors are processed in a variety of ML libraries based on neural networks.

It is worth noting that all the experimental results provided in this paper are based on approximations of one or multiple *K*'s intersecting all training samples or special label groups only in one direction. The preliminary results indicate the promise of the SVBF approach for finding a representative subspace for the set of datasets in classification tasks. The natural path forward is to increase the number of intersections in the SVBF optimization problem, i.e., increase the number of angles being used in the optimization problem. We also plan to explore this approach on more realistic data sets and explore how this impacts our ability to determine optimal *K*. We anticipate the main challenges will be in parallelizing the code efficiently to make larger problems computationally feasible.

## Data availability statement

The original contributions presented in the study are included in the article/supplementary material, further inquiries can be directed to the corresponding author.

## Author contributions

KK: Writing—original draft, Writing—review & editing. MK: Writing—original draft, Writing—review & editing. CP: Writing—original draft, Writing—review & editing.
